# Reducing the Antigen Prevalence Target Threshold for Stopping and Restarting Mass Drug Administration for Lymphatic Filariasis Elimination: A Model-Based Cost-effectiveness Simulation in Tanzania, India and Haiti

**DOI:** 10.1093/cid/ciae108

**Published:** 2024-04-25

**Authors:** Mary Chriselda Antony Oliver, Matthew Graham, Katherine M Gass, Graham F Medley, Jessica Clark, Emma L Davis, Lisa J Reimer, Jonathan D King, Koen B Pouwels, T Déirdre Hollingsworth

**Affiliations:** Department of Applied Mathematics and Theoretical Physics, University of Cambridge, Cambridge, United Kingdom; Big Data Institute, Li Ka Shing Centre for Health Information and Discovery, University of Oxford, Oxford, United Kingdom; Big Data Institute, Li Ka Shing Centre for Health Information and Discovery, University of Oxford, Oxford, United Kingdom; Neglected Tropical Diseases Support Centre, The Task Force for Global Health, Decatur, Georgia, USA; Centre for Mathematical Modelling of Infectious Disease and Department of Global Health and Development, London School of Hygiene & Tropical Medicine, London, United Kingdom; School of Biodiversity, One Health and Veterinary Medicine, University of Glasgow, Glasgow, United Kingdom; Mathematics Institute and the Zeeman Institute for Systems Biology and Infectious Disease Epidemiological Research, University of Warwick, Coventry, United Kingdom; Department of Vector Biology, Liverpool School of Tropical Medicine, Liverpool, United Kingdom; Department of Control of Neglected Tropical Diseases, World Health Organization, Geneva, Switzerland; Health Economics Research Centre, Nuffield Department of Population Health, University of Oxford, Oxford, United Kingdom; Big Data Institute, Li Ka Shing Centre for Health Information and Discovery, University of Oxford, Oxford, United Kingdom

**Keywords:** Lymphatic filariasis, modeling, elimination, threshold, health economics

## Abstract

**Background:**

The Global Programme to Eliminate Lymphatic Filariasis (GPELF) aims to reduce and maintain infection levels through mass drug administration (MDA), but there is evidence of ongoing transmission after MDA in areas where *Culex* mosquitoes are the main transmission vector, suggesting that a more stringent criterion is required for MDA decision making in these settings.

**Methods:**

We use a transmission model to investigate how a lower prevalence threshold (<1% antigenemia [Ag] prevalence compared with <2% Ag prevalence) for MDA decision making would affect the probability of local elimination, health outcomes, the number of MDA rounds, including restarts, and program costs associated with MDA and surveys across different scenarios. To determine the cost-effectiveness of switching to a lower threshold, we simulated 65% and 80% MDA coverage of the total population for different willingness to pay per disability-adjusted life-year averted for India ($446.07), Tanzania ($389.83), and Haiti ($219.84).

**Results:**

Our results suggest that with a lower Ag threshold, there is a small proportion of simulations where extra rounds are required to reach the target, but this also reduces the need to restart MDA later in the program. For 80% coverage, the lower threshold is cost-effective across all baseline prevalences for India, Tanzania, and Haiti. For 65% MDA coverage, the lower threshold is not cost-effective due to additional MDA rounds, although it increases the probability of local elimination. Valuing the benefits of elimination to align with the GPELF goals, we find that a willingness to pay per capita government expenditure of approximately $1000–$4000 for 1% increase in the probability of local elimination would be required to make a lower threshold cost-effective.

**Conclusions:**

Lower Ag thresholds for stopping MDAs generally mean a higher probability of local elimination, reducing long-term costs and health impacts. However, they may also lead to an increased number of MDA rounds required to reach the lower threshold and, therefore, increased short-term costs. Collectively, our analyses highlight that lower target Ag thresholds have the potential to assist programs in achieving lymphatic filariasis goals.

Lymphatic filariasis (LF), a debilitating neglected tropical disease caused by parasitic filarial worms transmitted through mosquitoes, has affected about 882 million people in 44 countries [1]. The World Health Organization's (WHO) 1997 proposal of LF elimination prompted the inception of the Global Program to Eliminate Lymphatic Filariasis (GPELF) in 2000 whose main objective was to achieve elimination of LF as a public health problem across 73 endemic nations by 2020 [2]. To date, WHO reports 19 countries and territories being validated as achieving elimination of LF as a public health problem, including Bangladesh and Lao People's Democratic Republic [1]. By 2021, 11 additional countries had successfully implemented recommended strategies, stopped large-scale treatment, and are under surveillance to demonstrate that elimination has been achieved [1].

The core intervention to reduce infection levels below a target threshold is annual mass drug administration (MDA) for ≥5 years in affected areas, along with vector control where possible. MDA uses either diethylcarbamazine + albendazole (termed DA) or albendazole + ivermectin (termed IA) [2], with some areas using the triple combination of ivermectin + diethylcarbamazine + albendazole (termed IDA) [3, 4]. To measure the impact of MDA and determine whether levels of infection have decreased below the threshold, WHO recommends sentinel and spot-check community surveys (“epidemiological monitoring surveys”), followed by a transmission assessment survey (TAS) [2], which is used to evaluate programs and inform decisions for stopping and restarting MDA (Figure 1*A* ).

**Figure 1.**
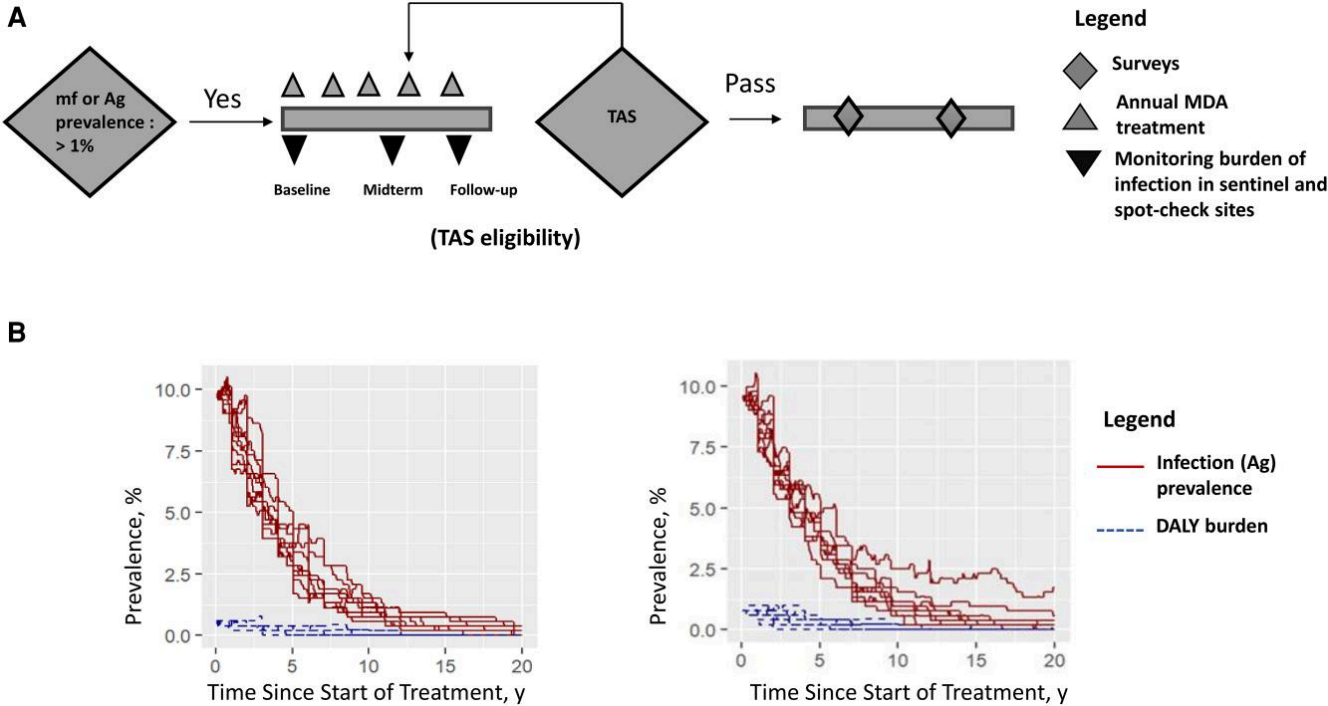
*A*, Schematic diagram representing the steps taken by the Global Program to Eliminate Lymphatic Filariasis to interrupt transmission of lymphatic filariasis (adapted from [2]). *B*, Timeline plots for illustrating the model-predicted temporal trends in antigenemia (Ag) prevalence (*solid lines*), disability-adjusted life year (DALY) burden are computed as the morbidity prevalence of lymphedema, hydrocele and acute adenolymphangitis (*dotted lines*) times the disability weights [5] for a low baseline prevalence (5%–10%) using <1% threshold (*left*) and <2% threshold (*right*) as the stopping threshold criteria for transmission assessment survey (TAS) with 80% mass drug administration (MDA) coverage of the total population. Abbreviation: mf, microfilaremia.

The TAS is a spatially representative survey of the population in an evaluation unit (EU; approximately <500 000 people) using the Alere Filariasis Test Strip. The survey design is built on the understanding that antigen-positive young children represent incident infection. Any positive signal in young children aged 6–7 years is cause for concern; infection levels in this age group should be very low if the program has achieved its aims of eliminating LF as a public health problem. The TAS, therefore, evaluates whether the upper 95% confidence interval (CI) for the prevalence of antigenemia (Ag) in this age group is less than the 2% target threshold. The exact sample size is guided by the characteristics of an EU, but approximately 30 sites are normally sampled, with 40–60 children 6–7 years old in each site. This results in a critical cutoff of <19 antigen-positive children for the EU to be declared as having “passed” the TAS and able to progress to posttreatment surveillance [2].

However, given the fact that the TAS relies on adult worm antigen detection as opposed to the presence of circulating microfilariae to indicate infection, and considering that the clearance of antigens lags behind that of microfilariae, relying on Ag in children may not be the optimal method to assess the effectiveness of IDA [6]. In countries where IDA is implemented, the WHO-recommended target threshold is <1% microfilariae threshold in adults (as per the IDA Technical Meeting Report, Coalition for Operational Research on Neglected Tropical Diseases (*COR-NTD*), October 2022).

Despite the success of MDA and the progress made by GPELF over the last decades, many challenges remain in achieving and sustaining LF elimination. For example, in certain regions, particularly where *Culex* mosquito is the main transmitting vector, there are difficulties in attaining [7] and maintaining the goals of the program [8, 9], possibly due to higher transmission efficiency by this vector [10–12]. There is also evidence of ongoing transmission in *Culex* areas that have passed the TAS, including in Sri Lanka [13] and Haiti [14], possibly due to the vector's increased competence [15–18]. One approach being considered to circumvent this problem is a reduction in the upper 95% CI of the threshold to <1% Ag prevalence (representing a critical cutoff of <7 antigen-positive children). Lower thresholds for stopping MDAs generally mean a higher probability of local elimination, reducing long-term costs and health impacts. However, they may also lead to an increased number of MDA rounds required to reach the lower threshold and, therefore, increased short-term costs.

To investigate these issues, here we use mathematical models of the transmission dynamics of LF to assess the potential implications of modifying the threshold for TAS. The central objective is to address a key policy question: What impact would ensue from reducing the TAS threshold, transitioning from <2% Ag prevalence (measured through the upper CI of the estimate, ie, the number of positive children with the critical cutoff being <19 for the standard design) to <1% Ag prevalence (measured as the number of positive children with the critical cutoff being <7)? Importantly, we focus on areas where the main transmission vector is *Culex* and for which there are documented country-specific cost-effectiveness thresholds, thereby selecting India, Haiti, and Tanzania. Across these, the main treatment strategy is DA (India and Haiti) and IA (Tanzania, due to coendemicity with onchocerciasis). We note that another article [19] in this supplement focuses on the use of the 3-drug combination IDA, which has particular challenges for survey design because of reductions in microfilariae density but not Ag over 1 or 2 rounds of treatment.

This study delineates 5 specific subquestions: (1) Does the reduction in the TAS threshold increase the positive predictive value (PPV) for local elimination? (2) Does the reduction in the threshold result in an increased number of MDA rounds and TAS surveys? (3) Does the reduction in the threshold contribute to a diminished health burden, specifically in terms of disability-adjusted life-years (DALYs) averted for morbidity? (4) How do the comparative costs and health impacts manifest between the prevailing and reduced thresholds? and (5) How to factor in the benefits associated with the likelihood of meeting GPELF targets in terms of the probability of local elimination by lowering the TAS target thresholds? Addressing these questions will help assess whether lower thresholds have the potential to assist programs in achieving LF local elimination goals and how such decisions affect program costs.

## METHODS

We use the stochastic TRANSFIL model [20] with the parameters previously estimated [21, 22] to represent transmission by *Culex* mosquitoes. The transmission model has been extended to include simulation of some health impacts of infection (see below). The transmission cycle starts with individual worms establishing in a host and then releasing immature microfilariae that circulate in the blood. These microfilariae are then picked up by the mosquitoes during blood feeding and transmitted to the next host when the next blood meal is taken. TRANSFIL captures the reduction in microfilariae in each host as an effect of MDA (see Box 1), as a function of simulated target coverage, systematic nonadherence, and the efficacy of the drugs used [24]. For the purpose of this study, we modeled only the impact of MDA and did not include other potential interventions, such as vector control.

Box 1. **Glossary. Source: Turner et al [23].**

1. Mass drug administration (MDA): A method of preventive chemotherapy involving the distribution of anthelminthic drugs to all eligible individuals within a specified area (such as a state, region, province, district, subdistrict, or village) on a routine basis, regardless of their individual infection status.
2. MDA coverage: The proportion of people who receive MDA.
3. Transmission assessment survey (TAS): A survey designed to measure whether evaluation units have lowered the prevalence of infection to a level where recrudescence is unlikely to occur, even in the absence of MDA interventions.
4. Disability-adjusted life years (DALYs): The number of years lost due to premature death of the disease and the years lived with disability caused by the disease.
5. Disability weights: A factor (between 0 and 1) that is used to calculate the number of years lost due to disability that accounts for the severity of the disease.
6. Worm burden: The quantity of filarial worms presents within a host organism, typically measured by the number or mass of worms within a specific anatomic location or system.
7. Morbidity: The prevalence or incidence of disease or illness within a population, often measured by the frequency and severity of health-related symptoms. In this study, *lymphedema* refers to the improper functioning of the lymph system that results in fluid collection and swelling; *hydrocele* refers to the swelling of the scrotum due to infection.
8. Willingness to pay (WTP): The amount of money the decision maker (individual, organization or government) would be willing to spend per unit of clinical effectiveness (in this case, DALYs averted for morbidity or 1% increase in probability of local elimination). In this study, we rely on the WTP per capita government expenditure for 3 different example countries.
9. Net monetary benefit (NMB): A summary statistic that represents the value of an intervention (in this case, stopping and restarting MDA) in monetary terms when a WTP threshold for a unit of benefit (DALYs averted for morbidity or 1% increase in probability of local elimination) is known.
10. Incremental NMB (INMB): The difference in NMB between alternative interventions, a positive INMB indicating that the intervention is cost-effective compared with the alternative at the given WTP threshold. In this case the incremental cost to derive the incremental benefit is less than the maximum amount that the decision maker would be willing to pay for this benefit.
11. Discounting: The process of adjusting future costs and outcomes to a present value. The discount rate determines the strength of the time preference.
12. Purchasing power parities: Rates of currency conversion eliminating differences in price levels between countries that are used to equalize the purchasing power of different countries.

We considered closed populations (ie, no migration, only births and deaths) of roughly 100 000–50 000 people representing the population size of an EU in a standard TAS, per the WHO guidelines [2] and an exponential age distribution. We simulated the detection of Ag using parameters which were fitted using bayesian Markov chain Monte Carlo framework to data from Malindi, Kenya, and Colombo and Gampaha, Western Sri Lanka [25]. MDAs were simulated with 65% and 80% coverage (see Box 1) of the total population, to simulate effective and high coverage. Systematic nonadherence to MDA is included in the model by calculating the individual probabilities of receiving the treatment based on the coverage and between-round correlation parameter which was parameterized against data fitted to Leogane, Haiti, and Egypt [24].

We extended the model to simulate the health impact of infection for the incidence of lymphedema, hydrocele and acute adenolymphangitis (ADL), based on previously published methods (section S1, part II, in the Supplementary Materials). Morbidity due to lymphedema and hydrocele was modeled by fitting the model to data from India [26]. The model assumes that damage sufficient to cause morbidity occurs once people have accrued a certain amount of damage due to a certain cumulative worm burden (see Box 1) in their lifetime. We further assume ADL to occur about twice per year (0–7 times) in 70% (45%–90%) of patients with hydrocele and 4 (0–7 times) times annually for 95% (90%–95%) of patients with lymphedema [27]. Prevalence of these morbid conditions was converted using published disability weights (see Box 1) for the symptoms of LF [5], following previous analyses in this area. In our model, we do not consider the adverse effects of MDA, despite records of 13% feeling unwell after MDA but able to do normal everyday activities and 3% reporting that they felt unwell enough that it stopped them doing normal everyday activities, such as going to school or work [28]. Despite evidence [29, 30] suggesting that mental illness is an underestimated dimension of the global disease burden for LF, limitations in crude estimates and a lack of accurate data of depressive illness in LF prevent its inclusion in our modeling calculations for DALY burden.

For replicating the starting and stopping decisions as prescribed by the WHO [2], we consider the TAS survey samples from 30 sites per EU. The distribution of baseline prevalences in these 30 sites is sampled from a normal distribution with 3 different means (eg, 5%–10%, 10%–20%, or 20%–30%). Therefore, we simultaneously simulate 30 closed sites and randomly sample 40–60 children aged 6–7 years from each of these sites to evaluate the TAS based on the critical cutoff. If the number of antigen-positive children across these 30 sites is greater than the critical cutoff, then we halt treatment until the next TAS survey. If the number of antigen-positive children does not exceed the critical cutoff then we continue MDA, per the algorithm (see Figure 1*A* ). We iterate the algorithm for 1000 runs and present the mean as the chosen baseline prevalences.

For simulating the costs, we consider them as weightings on the number of TAS surveys ($12 494.75 [31]) and number of MDA rounds ($7640.92 [32]) required for 3 example endemic countries—Tanzania, India ,and Haiti. The time horizon for running the model was selected as 20 years from the start of the program where discounting (see Box 1) has been included, in line with other analyses in this area. We highlight that for cost-effectiveness analysis, including when expressing the cost-effectiveness using the metric expected incremental net monetary benefit (INMB) (section S4, part II, in the Supplementary Materials for more details), we require a comparator that has been simulated using the TAS survey where children aged 6–7 years were sampled for Ag prevalence with the threshold of <2% Ag prevalence.

As defined in Box 1, for the willingness to pay (WTP) in US dollars $ per DALY averted for morbidity, we consider the per capita government expenditure, a country-specific cost-effectiveness threshold reflecting the opportunity costs—for Tanzania ($389.83 [33]), India ($446.07 [33, 34]), and Haiti ($219.84 [33])—which has been adjusted for the purchasing power parity [35]. Specifically, we made use of the provided country-specific percentage of GDP per capita estimate that underlies the DALY-4 estimation method by multiplying the total per individual DALY value times a specific proportion of the GDP per capita [33] for computing the WTP per DALY averted for morbidity. For valuing the gains from local elimination in order to align countries’ efforts to the GPLEF goals, we simulate for a range with fixed values of the WTP for local elimination ($0, $5000, $10 000) [36] per 1% increase in probability of local elimination.

In summary, we simulated the transmission dynamics and morbidity associated with LF, including DALY burden (section S2, part II, in the Supplementary Materials) for 30 sites and simulated a TAS-like survey across those sites for starting and stopping the MDA based on the different critical cutoffs. We investigated our modeling approach across different MDA coverages (65% and 80% of the total population) and different baseline LF prevalences. We considered the WTP for 3 countries for which culicine mosquitoes are major transmitters—India, Haiti, and Tanzania. We evaluated the impact of the different critical cutoffs using a model of the transmission dynamics, health and economic impact of LF. Therefore, to answer the 5 crucial questions outlined above which are pertinent to our study, we use the following intermediate outcomes: (1) PPV [10], the probability of achieving local elimination (ie, proportion of simulations having no new transmissions within 20 years after the last round of MDA) if the 1-year post-MDA Ag prevalence in a sample of <1700 children aged 6–7 years was below the given threshold; (2) evaluation of health impact through DALYs (see Box 1); (3) costs due to MDA rounds and TAS surveys; (4) comparison of critical cutoffs with their respective TAS target thresholds, using the cost-effectiveness approach with the help of the expected INMB metric (see Box 1).

## RESULTS

The impact of the infection on the interruption of LF transmission and reduction of the disease burden using DALYs depends on the threshold criteria defined for passing the TAS, as illustrated in the example of a setting with a baseline prevalence of 5%–10% and 80% MDA coverage of the total population (Figure 1*B* ).

**Figure 2.**
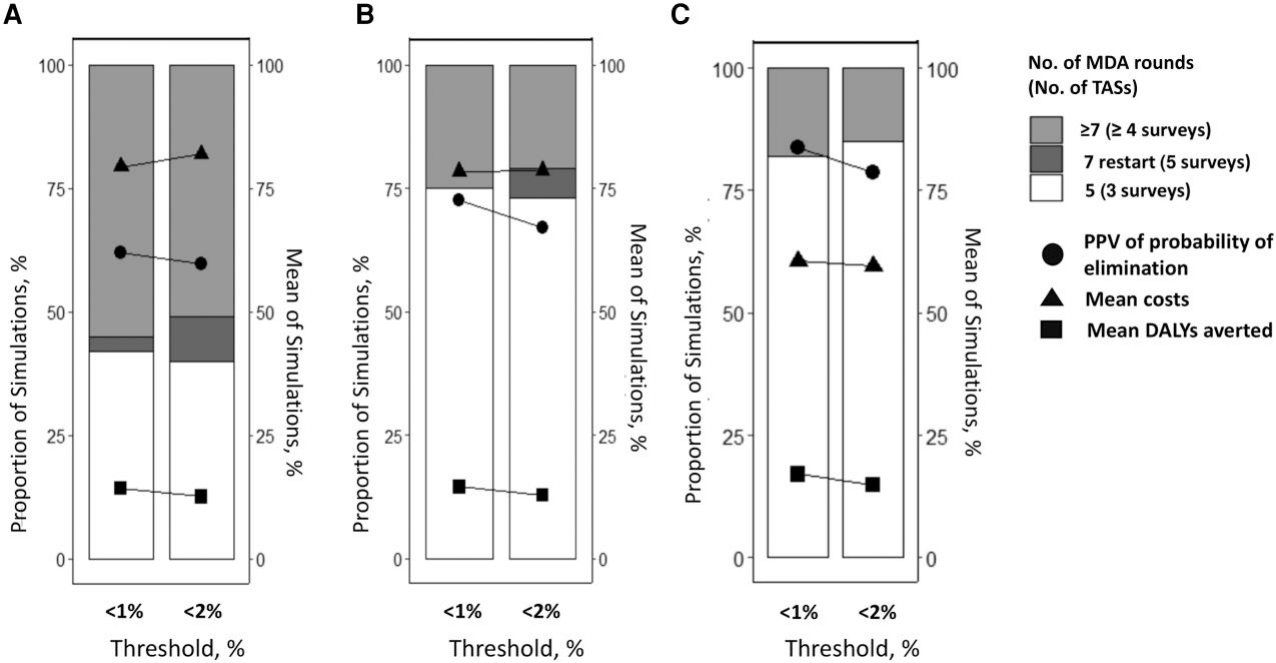
Stacked bar plot shows the trend in the number of rounds of mass drug administration (MDA) treatment and transmission assessment surveys (TASs) required for a sample of <1700 children aged 6–7 years with different antigenemia baseline prevalence—20%–30% (*A*), 10%–20% (*B*), 5%–10% (*C*)—for 80% MDA coverage of the total population simulated for the diethylcarbamazine + albendazole drug. Light gray represents ≥7 rounds; dark gray, 7 rounds with restarts; and white, 5 rounds. The secondary axis represents the mean of the simulations for the epidemiological outcomes. Circles represent positive predictive value (PPV) of the probability of local elimination; triangles, mean costs; and squares, mean disability-adjusted life-years (DALYs) averted for morbidity.

We find that the PPVs for local elimination were 83.8% (for 5%–10% baseline prevalence), 72.57% (for 10%–20% baseline prevalence), and 62.08% (for 20%–30% baseline prevalence) of the model simulations for a threshold of <1% Ag prevalence as the stopping criteria. Alternatively, for a threshold of <2% Ag prevalence as the stopping criteria, we find that PPV was achieved in about 78.8%, 67.15%, and 59.64% of the simulations, respectively (Supplementary Table 4 [part III in the Supplementary Materials]). Our findings, as highlighted in Figure 2 (circles), suggest that, regardless of the baseline prevalence and coverage, lowering the threshold increases the PPV for local elimination. These trends follow across different baseline prevalences, MDA coverages and treatment strategies (Supplementary Table 4 and 7 [part III for DA drug combination and part III for IA drug combination, respectively, in the Supplementary Materials]).

Figure 2 also illustrates that a lower threshold results in an increase in the proportion of simulations that require extra rounds to achieve the threshold. In general, a lower threshold leads to a reduction in the probability of having to restart after stopping (driven by the higher probability of local elimination), resulting in fewer MDA rounds and surveys (see Supplementary Table 6*A*, part III for DA and Supplementary Table 8*A*, part III for IA in the Supplementary Materials). Therefore, in general, lower costs are associated with the lower threshold (Figures 2*A* and 2*B* , triangles). However, for the lowest baseline prevalence simulations, restarting MDA is unlikely for either threshold (Figure 2*C* ), so the difference in mean costs is driven by the slightly higher proportion of simulations that require extra rounds to reach the lower threshold, resulting in higher mean costs for the lower threshold (Figure 2*C* , triangles). Similar trends are evident for 65% MDA coverage of the total population (see Supplementary Table 6*B*, part III for DA and Supplementary Table 8*B* for IA in the Supplementary Materials).

Figure 2 (squares) also depicts the mean DALYs averted per morbid condition across different thresholds with varying baseline prevalences for 80% MDA coverage of the total population with DA as a treatment strategy. Lowering the threshold causes a small but discernible change in the morbidity prevalence (as depicted in Figure 1*B* ), with more DALYs averted than with the higher threshold. This limited change occurs since for both thresholds the incidence of DALY burden drops dramatically as average worm burdens drop, so most morbidity prevalence is due to historic infection, before the MDA (see Supplementary Figure 2, part III for IA as a treatment strategy). Similar trends are evident for 65% MDA coverage of the total population (see Supplementary Figure 1, part III for DA and Supplementary Figure 3, part III for IA).

Since the DALY difference is relatively smaller than the gain in the mean costs due to the combined effect of the increase in the MDA rounds and TAS surveys at higher thresholds and/or baseline coverages, the choice of critical cutoff is highly dependent on the epidemiological context (baseline prevalence, coverage) and the economic considerations of the health impact using the WTP per DALY averted, which are usually fixed specific to the country under consideration.

**Figure 3.**
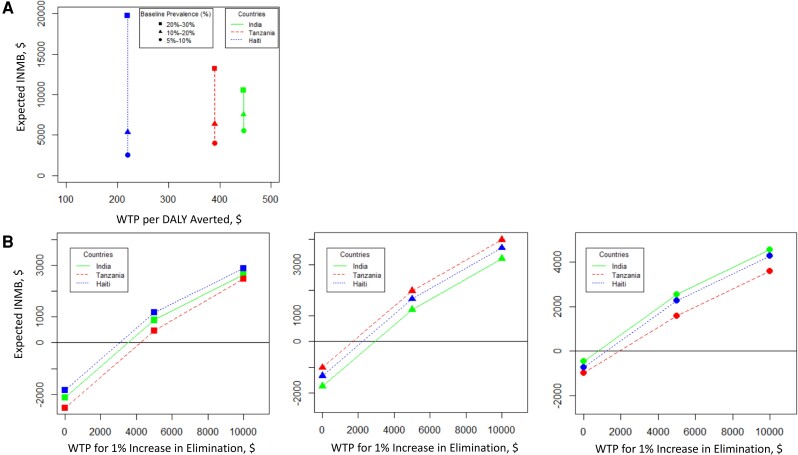
*A*, Expected incremental net monetary benefit (INMB, in dollars) of switching from <2% threshold to <1% threshold of the antigenemia (Ag) prevalence in the transmission assessment survey (TAS) for different baseline prevalence indicated by symbols (squares for 20%–30%, triangles for 10%–20%, and circles for 5%–10%) and different countries (India [diethylcarbamazine + albendazole (DA)], solid line; Tanzania [albendazole + ivermectin (IA)], dashed line; and Haiti [DA], dotted line) for the willingness to pay (WTP) per disability-adjusted life-year (DALY) averted without accounting for the benefits of local elimination using 80% mass drug administration (MDA) coverage of the total population. *B*, Expected INMB of switching from <2% threshold to <1% threshold of the Ag prevalence in the TAS for different baseline prevalence indicated by symbols (squares in the left for 20%–30%, triangles in the middle for 10%–20% and circles in the right for 5%–10%) and different countries (India [DA], solid line; Tanzania [IA], dashed line; Haiti [DA], dotted line) simulated for a fixed set of WTP ($0, $5000, and $10 000) per 1% increase in the probability of local elimination using 65% MDA coverage of the total population. The solid black horizontal line depicts the minimum WTP per unit increase in the probability of local elimination, such that <1% threshold is cost-effective for the 65% MDA coverage.

To comprehensively evaluate the joint considerations of costs, health impact, and the monetization benefits obtained from the probability of local elimination, we use the metric of expected INMB. A higher expected INMB signifies the optimal cost-effectiveness of a specific threshold compared with the alternative, given the WTP per DALY averted, a parameter that varies among different countries. Moreover, the analysis incorporates a WTP for local elimination, which is subject to variation based on the 1% increase in the probability of local elimination for the individual baseline and coverage. Our findings, as depicted in Figure 3*A* , indicate that if the effective coverage of the total population is 80%, then the switch to a lower threshold is cost-effective across all baseline prevalences; that is, the cost per DALY averted for morbidity over the 20-year program remains below the national threshold WTP (positive expected INMB; see Supplementary Table 5, part III in the Supplementary Materials).

The heterogeneous results within these countries for higher coverage for different baseline prevalence are largely due to demographic patterns that affect the DALY estimates [37], such as age composition, treatment strategy, life expectancy and population growth rates. Likewise, as shown in Figure 3*B* , for the relatively lower MDA coverage (65% of the total population), the increased number of rounds and surveys suggests that the switch to a lower threshold would only be considered based on the WTP per 1% increase in the probability of local elimination, thereby factoring in the premium of elimination that is imperative to bring a country's activities in line with GPELF goals. For example, for a WTP of approximately $4000, $2200, and $1000 per 1% increase in the probability of local elimination, for the 20%–30%, 10%–20%, and 5%–10% baseline prevalences studied, respectively, the analysis would recommend a switch to a lower threshold (see Figure 3*B* , solid black line).

## DISCUSSION

Stopping thresholds form a core element of several disease control policies and determine the probability of local elimination. However, it is worth investigating not only whether a lower threshold increases the probability of local elimination but also how it affects the overall costs of the program. This study draws attention to the potential benefits of a lower threshold, as exemplified by the implementation of such a lower threshold in China and its role in successful LF control. Importantly, China combined multiple interventions (including vector control) to arrest transmission and benefited from chemotherapy being based on the use of fortified salt, which enabled achieving nearly universal coverage since China controlled salt supplies and environmental vector management [38]. Nonetheless, this example provides important information for the GPELF to set criteria for evaluating whether MDA has succeeded in lowering the prevalence of infection to a level where recrudescence is unlikely to occur.

In the current study, we investigated the impact of different stopping thresholds for the model-derived dynamics in TAS, based on the survey results from 30 sites. This is a major simplification but leads to insight before investigating the effect of spatial heterogeneity or migration in and out of communities. We further assume that the change in threshold would not result in a change to the survey design so that the impact of the change in threshold in MDA rounds and a number of surveys could be considered separately from any change in the cost of the survey.

Our findings highlight that reducing the Ag prevalence threshold from <2% to <1% increases the probability of local elimination. We also show that while a lower threshold results in an increase in the proportion of simulations that require extra rounds to achieve the threshold, it also generally reduces the probability of having to restart after stopping. However, at the lowest prevalence, there is a low probability of restarting at either threshold, and therefore the mean costs associated with reducing the threshold could be slightly higher due to the small probability of having to perform additional rounds to reach the threshold. Reducing the threshold also reduces the DALY burden over the course of the program, but this gain is modest, given the ongoing low prevalence and intensity of infection (and therefore incident morbidity) during treatment programs for both thresholds.

Our analysis also evaluates the joint considerations of costs, health impact, and the monetization benefits obtained from the probability of local elimination using the expected INMB metric. We find that the switch to a lower threshold is cost-effective across all baseline prevalences for our 3 example countries unless MDA coverage is relatively lower (65%) than 80% MDA coverage. We further extended the model to factor in the premium of elimination that is imperative to bring a country's activities in line with GPELF goals for an additional WTP of approximately $4000 (for 20%–30% baseline prevalence), $2200 (for 10%–20% baseline prevalence), and $1000 (for 5%–10% baseline prevalence) per 1% increase in the probability of local elimination. Therefore, the analysis would recommend that a switch to a lower threshold would be cost-effective with 65% MDA coverage (see Figure 3*B* , solid black line).

Modeling morbidity for LF is challenging due to the dependence of the estimates on the Global Burden of Disease study [5], which are notably uncertain given the paucity of data on their geographic spread and control [39]. By tracking morbidity only for hydrocele, lymphedema and ADL, but not other outcomes such as mental health impacts [29, 30, 39], lymphatic dilation, and tropical pulmonary eosinophilia or the potential for productivity losses due to illness (either due to lack of data) [40], we underestimate the true burden of disease. Because of the lack of data for the systematic nonadherence correlation in our setting, we use the values from the data analyzed by Dyson et al [24]. They note that for a range of MDA coverage from 40.5% to 95.5%, the correlations found lie in a fairly narrow range (between 0.2806 and 0.5351), indicating that the level of systematic nonadherence may be similar even in data for different years, countries, diseases, and administered drugs [24].

Another critical part of the design of this study is that the survey implementation costs remain the same so are not considered across different survey designs. Hence, the costs of MDAs and TAS do not factor in out-of-pocket expenses that might have a detrimental macroeconomic impact [41–43] and may change over the years to come. Several studies [44–46] quantifying the costs for MDA and TAS show that investigators encountered difficulties in estimating the precise allocation of time and resources to LF MDA programs due to the lack of previous tracking, incomplete usage records, challenges in accessing accurate cost data from government sources, and potential underreporting or misallocation of costs despite efforts to validate reported expenses with international partners and nongovernmental organizations [47]. However, these simulations are a useful tool for understanding the impact of TAS thresholds on stopping MDA using the dynamics and impacts of this infection.

Another limitation of our analysis is that it focuses exclusively on MDA and does not account for the potential benefits provided by additional interventions, such as vector control. We decided not to include vector control in our simulations since its benefits in regions where *Culex* is the main transmission vector remain the subject of debate. For example, some studies have shown that vector control in combination with MDA may be beneficial to reduce the mosquito biting rates [20] in low-endemic settings, such as Tanzania [48] and India [49]. However, other analyses [12, 50] have shown that vector control did not enhance the likelihood of achieving transmission elimination or reduced the probability of recrudescence compared with MDA alone, whether during the 5-year TAS period or over a longer 20-year period. Future studies are needed to further clarify the potential benefits provided by vector control in these settings.

Despite these limitations, our study underscores the significance of selecting appropriate criteria for decision making, particularly in the context of LF interventions. It is vital to determine whether LF interventions effectively halt disease transmission in endemic communities. Simultaneously, understanding the dynamics of local elimination after threshold crossing and its interaction with LF interventions is crucial. Our work highlights that the path to LF local elimination after MDA surveillance is gradual, involving a prolonged transient phase, but that lower thresholds have the potential to assist programs in achieving their goals.

## Supplementary Data

Supplementary materials are available at *Clinical Infectious Diseases* online. Consisting of data provided by the authors to benefit the reader, the posted materials are not copyedited and are the sole responsibility of the authors, so questions or comments should be addressed to the corresponding author.

## Supplementary Material

ciae108_Supplementary_Data

## Notes

***Author contributions.*** M. C. A. O. performed the analysis and wrote the draft of the paper. K. B. P. and T. D. H. supervised the work. M. G. provided the transmission model and consulted on the details of the simulation approach. K. M. G., L. J. R., K. B. P., and T. D. H. conceived the more specific work in discussion with coauthors, who all contributed to the analysis and reviewed the draft.

***Disclaimer.*** The funders had no role in study design, data collection and analysis, decision to publish, or preparation of the manuscript.

***Supplement sponsorship.*** This article appears as part of the supplement “New Tools and Nuanced Interventions to Accelerate Achievement of 2030 Roadmap for Neglected Tropical Diseases,” sponsored by funding of Professor T. Déirdre Hollingsworth's research by the Li Ka Shing Foundation at the Big Data Institute, Li Ka Shing Centre for Health Information and Discovery, University of Oxford; and funding of the NTD Modelling Consortium by the Bill & Melinda Gates Foundation (INV-030046).

***Financial support.*** This work was supported by the Bill & Melinda Gates Foundation (INV-030046), via the NTD Modelling Consortium; the Health Data Research UK-The Alan Turing Institute Wellcome (grant 218529/Z/19/Z to M. C. A. O.); and the Commonwealth European and International Trust (Cambridge Trust scholarship to M. C. A. O.).
