# Supplementary material for: Reducing the Antigen Prevalence Target Threshold for Stopping and Restarting Mass Drug Administration for Lymphatic Filariasis Elimination: A Model-Based Cost-effectiveness Simulation in Tanzania, India and Haiti
Source: Clin Infect Dis. 2024 Apr 25;78(Suppl 2):S160–8. doi: 10.1093/cid/ciae108 (PMC11045020; doi:10.1093/cid/ciae108)
Supplement: ciae108_Supplementary_Data [file ciae108_supplementary_data.docx]

**Supplementary Information - Methodology**

Part I. Parameter Values

**Table S1:** Parameter values used in TRANSFIL [20,21] simulations, taken from previously published model documentation if not otherwise stated.

| Parameter | Value/Scenario |
| --- | --- |
| Baseline mf prevalence (%) | 5 -10% ,  10-20%,  20-30% |
| Drug | DA, IA |
| MDA frequency | Annual |
| MDA coverage | 65%,80% |
| MDA systematic non-adherence correlation | 0.2806 - 0.5351 |
| EPHP threshold (Ag prevalence) | <1%, <2% |
| Primary vector species | Culex |
| Bite risk aggregation parameter, ($\boldsymbol{k}$) | 0.01 – 0.1 |
| Annual biting rate (ABR) | 0 – 1200 |
| Vector control coverage | 0, 0.5, 0.8 |
| Insecticidal decay half-life | 2 years |
| Bite rate per mosquito per month ($\boldsymbol{\lambda}$) | 5-15 |
| Proportion of mosquitoes infected by infectious bite | 0.37 |
| L3 uptake and development parameter ($\boldsymbol{\kappa}$) | 4.395 |
| L3 uptake and development parameter ($\boldsymbol{r}_{\boldsymbol{1}}$) | 0.055 |
| Mosquito death rate per month | 5 |
| Mf birth rate per female worm per month ($\boldsymbol{\alpha}$) | 1 |
| Proportion L3 leaving mosquito per bite ($\boldsymbol{\psi}_{\boldsymbol{1}}$) | 0.414 |
| Proportion L3 leaving mosquito that enter host ($\boldsymbol{\psi}_{\boldsymbol{2}}$) | 0.32 |
| Proportion L3 entering host that develop to adults ($\boldsymbol{s}_{\boldsymbol{2}}$) | 0.00275 |
| Adult worm death rate per month ($\boldsymbol{\mu}$) | 0.0104 |
| Mf death rate per month ($\boldsymbol{\gamma}$) | 0.1 |
| Host death rate per month ($\boldsymbol{\tau}$) | 0.00167 |
| Proportion of mf killed by IA treatment | 0.99 |
| Proportion of adult worms killed by IA treatment | 0.35 |
| Length of worm sterilisation after IA treatment (months) | 9 |
| Reduction in individual bite risk in presence of LLINs (efficacy) | 0.97 |
| Shape parameter for gamma distribution (lymphoedema - gl ) | 0.02 |
| Shape parameter for gamma distribution (hydrocele - gh) | 0.71 |

**Table S2:** Parameter values used in calculating the epidemiological outcomes, taken from previously published documentation if not otherwise stated.

| Parameter | Value/Scenario |
| --- | --- |
| Weights for the TAS surveys [30]  Weights for the MDA rounds [31]  Number of simulations | 12494.75 ($)  7640.92 ($)  1000 |
|  |  |

Remark: All the fixed costs are considered in the US dollars ($), adjusted for the purchasing power parity (PPP) [34].

**Table S3:** Parameter values used in calculating the cost-effective framework, taken from previously published documentation if not otherwise stated.

| Parameter | Value/Scenario |
| --- | --- |
| Willingness to pay for DALYs averted for morbidity (India) [32,33]  Willingness to pay for DALYs averted for morbidity (Tanzania) [32]  Willingness to pay for DALYs averted for morbidity (Haiti) [32] | 446.07 ($)  389.83 ($)  219.84 ($) |
|  |  |

Remark: All the costs are considered in the US dollars ($), adjusted for the purchasing power parity (PPP) [34].

Part II. Methodology for calculating the epidemiological outcomes

**S1.Calculation of the prevalence of morbidity (hydrocele, lymphoedema and ADL)**

Each individual is assigned a susceptibility to having hydrocele (if female, this = 0) and lymphoedema. These susceptibilities are drawn from a gamma distribution with a specific shape gl and rate 1/gl (giving the distribution a mean of 1). This susceptibility is drawn at birth and is never changed for the individual. To assess if someone has one of these sequelae, we multiply their susceptibility to the sequelae by the total worms they have had over their lifetime. If this value is greater than a chosen number, then they will be designated as displaying this sequela. A consequence of this is that once a person has a sequela, they will always have it.

For lymphoedema the shape parameter is 0.0033 and for hydrocele it is 0.02 [24]. The number they are compared to is the same for both at 11.3 which was estimated as a fit to a dataset from India [24].

We further assume ADL to occur about twice per year (0–7 times) in 70% (45– 90%) of hydrocele patients, and four (0–7 times) times annually for 95% (90–95%) of patients with lymphoedema [25].

**S2.Calculation of the DALY averted for morbidity**

In order to estimate the burden of morbidity caused by lymphatic filariasis, we propose the following DALY framework. The burden of the disease can be mathematically estimated as follows,

$$DALY =YLL+YLD$$

where YLL is the years lost due to premature death of the disease and YLD is the years lived with disability caused by the disease. We modified this mathematical formula for the estimating the burden of morbidity caused by filariasis as a cause. Consequently, we compute the two components of DALYs as follows:

$$YLD=P*dw$$

where P is the prevalence of morbidity (computed as mentioned in Part II, subsection S1.) and dw is the published disability weights for lymphoedema and hydrocele [26].

$$YLL=N*L$$

where N is the number of deaths caused by the burden of the disease and L is the standard expected life expectancy at the age of death. According to GBD, no deaths occurred as a result of the burden of morbidity caused by lymphatic filariasis. In addition, according to the WHO, although filariasis is one of the leading causes of disability, death from filariasis is rare. Therefore, we will assume N~ 0. This in turn leads to the years lost due to premature death as zero. Hence, the estimated DALY burden due to morbidity is simply the prevalence of morbidity (computed from Part II, subsection S1.) times the disability weights [26]:

$$DALY =YLD=P*dw$$

Therefore, the DALYs averted is computed as,

$$DALYs averted=DALY burden before starting MDA-DALY burden after stopping MDA$$

**S3. Calculation of the total costs**

The total costs are computed using the proposed mathematical equation.

$$Total costs=w_{1}*Total MDA rounds+w_{2}*Total TAS surevys conducted$$

Where w1 [31] and w2 [30] are the estimated cost weightings computed in accordance with the current purchasing power parity in the US $. The total MDA rounds and total TAS surveys conducted are computed from the TRANSFIL simulations by keeping a counter for each simulation under the different scenarios. The expected estimated cost weightings of MDA [31] and TAS surveys [30] (excluding the costs for rapid diagnostic tests RDT) for LF programs with annual treatment from the perspective of the endemic country government cover financial and economic costs. The financial costs are the costs of all inputs purchased in cash for MDA, including purchased MDA drugs, materials and supplies, ministry of health personnel salaries, and per diem payments for community drug distributors. Economic costs also include the costs of donated drugs for MDA

**S4. Calculation of the expected incremental net monetary benefit (INMB)**

The total costs are determined by weights associated with the number of surveys and the DALYs (prevalence of morbidity). These projected costs alongside the DALYs are used to decide the optimal surveillance design for post-MDA surveillance. The decision to choose a particular strategy for surveillance design is based on the expected utility theory of selecting the maximum utility attached with each surveillance design thereby reducing the total costs after the cessation of the MDA treatment. These decisions are typically made using the net monetary benefit which is obtained by maximizing the expected net monetary benefits. These net monetary benefits are derived by averaging over the population and the given parameters. For a given subgroup g (in this case sample of children aged 6-7 years old), and parameter set θ, NMBs are computed as the difference between the monetized health gains from an intervention less costs, or,

$$NMB_{g}\left( j,\theta\right)=e_{gj}.k-c_{gj}$$

where $e_{gj}$ and $c_{gj}$ are the measures of clinical effectiveness (e.g. DALYs) and costs in subgroup g, using threshold criteria j, respectively, and k is the decision makers willingness to pay (WTP) per unit of clinical effectiveness. The optimal threshold criteria for a given subgroup is the one that maximizes expected NMBs,

$$j_{g}^{*}=argmax_{j}E_{\theta}\left( NMB_{g}\left( j,\theta\right) \right)$$

This in general is then compared relative to a standard threshold criterion which is known as the “comparator”. In this scenario the <2% threshold for the individual baseline prevalence and MDA coverage will be considered as the comparator. This can be explained with an example – for instance, threshold criteria <1% is preferred over <2% in a particular subgroup g if $E_{\theta}\left( INMB_{\theta} \right)>0$, where for a particular subgroup g

$$INMB_{\theta}\left( \theta\right)=NMB_{g}\left( j=i,\theta\right)-NMB_{g}\left( j=<2\%,\theta\right) \forall i=<1\%$$

$$k>\frac{E_{\theta}(c_{gi}-c_{g0})}{E_{\theta}(e_{gi}-e_{g0})}=ICER_{g}$$

There are three additional cases:

- Threshold i, *dominates* threshold <1%  if it is more effective and less costly.
- Threshold i, is *dominated* by threshold <1% if it is less effective and more costly.
- Threshold i, is preferred to threshold <1% if it is less effective and less costly when k< $ICER_{g}$.

The cost-effectiveness plane plots the incremental effectiveness of a threshold criteria (relative to a comparator) against the incremental costs associated. The plot is useful because it demonstrates both the uncertainty and the magnitude of the estimates. Each point on the plot is from a particular value estimated from the TRANSFIL model. For each sampled parameter set and threshold criteria, the estimate differences in costs and DALYs relative to the comparator. For a given WTP based on different countries (as estimated in Part I, Table III), the expected INMB are computed by averaging over the proportion of the simulations which by default are represented for 95% confidence intervals. The higher the expected INMB is, the most cost-effective is the threshold.

Part III. Supplementary findings for varying baseline prevalences and coverages

The table below summarises the trend across the thresholds based on the positive predictive value for elimination across different baseline prevalences and MDA coverages.

**Table S4: Positive predictive value for elimination (i.e. percentage of simulations for which elimination occurs by the end of 20 years after starting MDA) for different baseline prevalences with 80%,65% MDA coverage of the total population for DA drug.**

|  |  | Critical cutoff (target threshold with upper 95% CI) | |
| --- | --- | --- | --- |
| MDA coverage | Mean baseline prevalence | <7 antigen-positive children (<1%) | <19 antigen-positive children (<2%) |
| 80%  65% | 5-10% | 83.8% (83.79 - 83.82)  78.19% (78.12 - 79.02) | 78.8% (78.79 - 78.80)  72.7% (72.69-72.70) |
| 80%  65% | 10-20% | 72.57% (70.56 - 73.58)  71.25% (71.23-71.26) | 67.15% (64.13 - 68.16)  65.34% (64.36 - 65.41) |
| 80%  65% | 20-30% | 62.08% (57.54 - 65.35)  61.45% (60.54 - 64.35) | 59.64% (55.63 - 59.68)  58.23% (57.63 - 59.67) |

The table below summarises the results for the expected incremental net monetary benefit (in dollars) of switching from less than 2% threshold to less than 1% threshold in the TAS for each setting (different countries, different baseline prevalences and different MDA coverages), with different willingness to pay for DALYs averted for morbidity and willingness to pay for per unit increase in the probability of elimination.

**Table S5: Expected incremental net monetary benefit (in dollars) of switching from <2% threshold to <1% threshold in the TAS for each setting, not accounting for willingness to pay for elimination. Note: The comparator for computing the INMB is the <2% threshold (critical cut off being <19 antigen positive) in 6-7 years old for the individual baseline prevalence and MDA coverage** **of the total population. This has been chosen for the three countries India and Haiti (IA drug) and Tanzania (DA drug) so that we can estimate the cost-effectiveness of the threshold at different known baseline prevalence and MDA coverage.**

| Baseline  Prevalence | Country (WTP per DALY averted) | | | | | |
| --- | --- | --- | --- | --- | --- | --- |
|  | India ($ 446.07) | | Tanzania ($ 389.83) | | Haiti ($ 219.84) | |
|  | MDA Coverage | | MDA Coverage | | MDA Coverage | |
|  | 80% | 65% | 80% | 65% | 80% | 65% |
| Willingness to pay $0 per 1% increase in the probability of elimination to switch thresholds | | | | | | |
| 5-10% | $5,556 | -$4,55 | $4,009 | -$9,64 | $2,536 | -$7,39 |
| 10-20% | $7,525 | -$1,755 | $6,381 | -$1,023 | $5,369 | -$1,334 |
| 20-30% | $10,573 | -$2,121 | $13,223 | -$2,538 | $19,744 | -$2,634 |
| Willingness to pay $10,000 per 1% increase in the probability of elimination to switch thresholds | | | | | | |
| 5-10% | $5,556 | $4,545 | $4,009 | $3,586 | $2,536 | $4,261 |
| 10-20% | $7,525 | $3,248 | $6,381 | $3,977 | $5,369 | $3,662 |
| 20-30% | $10,573 | $2,642 | $13,223 | $2,489 | $19,744 | $2,876 |
| Minimum willingness to pay $5,000 per 1% increase in probability of elimination to switch thresholds | | | | | | |
| 5-10% | $5,556 | $2,545 | $4,009 | $1,586 | $2,536 | $2,261 |
| 10-20% | $7,525 | $1,248 | $6,381 | $1,977 | $5,369 | $1,666 |
| 20-30% | $10,573 | $879 | $13,223 | $4,62 | $19,744 | $1,036 |

**Table S6 (a): Total number of MDA rounds and TAS surveys (i.e. proportion of simulations that achieve these rounds) for different baseline prevalences with 80% MDA coverage for the DA drug.**

|  |  | Critical cutoff (target threshold with upper 95% CI) | |
| --- | --- | --- | --- |
| Mean baseline prevalence | Total Number of MDA rounds (TAS surveys) | <7 antigen-positive children (<1%) | <19 antigen-positive children (<2%) |
| 5-10% | 5 rounds (3 surveys)  >= 7 rounds (4 surveys)  7 restart (5 surveys) | 82%  18%  0% | 85%  15%  0% |
| 10-20% | 5 rounds (3 surveys)  >= 7 rounds (4 surveys)  7 restart (5 surveys) | 78%  22%  0% | 76%  20%  4% |
| 20-30% | 5 rounds (3 surveys)  >= 7 rounds (4 surveys)  7 restart (5 surveys) | 47%  50%  3% | 45%  46%  9% |

**Table S6 (b): Total number of MDA rounds and TAS surveys (i.e. proportion of simulations that achieve these rounds) for different baseline prevalences with 65% MDA coverage for the DA drug.**

|  |  | Critical cutoff (target threshold with upper 95% CI) | |
| --- | --- | --- | --- |
| Mean baseline prevalence | Total Number of MDA rounds (TAS surveys) | <7 antigen-positive children (<1%) | <19 antigen-positive children (<2%) |
| 5-10% | 5 rounds (3 surveys)  >= 7 rounds (4 surveys)  7 restart (5 surveys) | 76%  24%  0% | 80%  20%  0% |
| 10-20% | 5 rounds (3 surveys)  >= 7 rounds (4 surveys)  7 restart (5 surveys) | 68%  30%  2% | 72%  24%  4% |
| 20-30% | 5 rounds (3 surveys)  >= 7 rounds (4 surveys)  7 restart (5 surveys) | 39%  52%  9% | 41%  46%  13% |

**Figure S1. Stacked bar plots illustrating the proportion of simulations with the total number of MDA rounds and TAS surveys required (dark grey : more than 7 rounds with restarts, light grey : greater than 7 rounds, white : 5 rounds) across different thresholds with varying baseline prevalences at 65% MDA coverage of the total population alongside the epidemiological outcomes for the DA drug**.


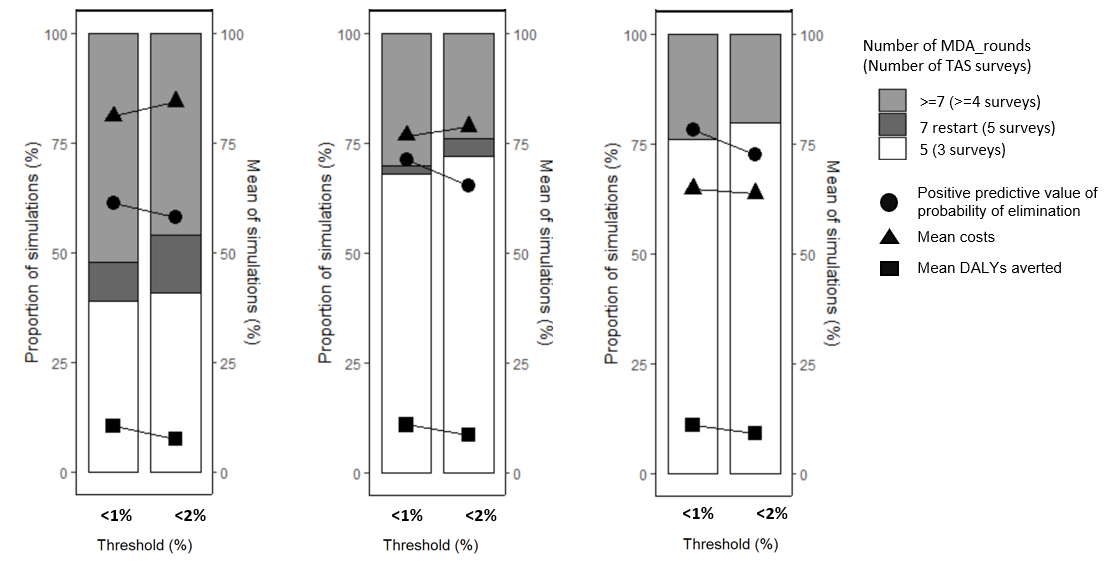


*Figure S1:* *Stacked bar plot shows the trend in the number of rounds of MDA treatment and TAS surveys required for a sample of less than 1700 children aged 6-7 years old with different Ag baseline prevalence (20-30% - left, 10-20% - middle, 5-10% - right) for 65% MDA coverage of the eligible population using DA drug. Light grey represents greater than equal to 7 rounds, dark grey represents 7 rounds with restarts and white represents 5 rounds.  The secondary axis represents the mean of the simulations for the epidemiological outcomes – Circles for positive predictive value of the probability of elimination, Triangles for the mean costs and Squares for the mean DALYs averted for morbidity.*

**Table S7: Positive predictive value for elimination (i.e. percentage of simulations for which elimination occurs by the end of 20 years after starting MDA) for different baseline prevalences with 80%,65% MDA coverage of the total population for IA drug.**

|  |  | Critical cutoff (target threshold with upper 95% CI) | |
| --- | --- | --- | --- |
| MDA coverage | Mean baseline prevalence | <7 antigen-positive children (<1%) | <19 antigen-positive children (<2%) |
| 80%  65% | 5-10% | 76.2% (74.79 - 78.24)  71.4% (70.34 – 73.23) | 72.3% (70.2 - 74.58)  67.5% (65.54 - 69.63) |
| 80%  65% | 10-20% | 68.57% (67.36 - 70.38)  62.3% (61.12- 65.24) | 62.15% (60.13 - 63.26)  59.4% (57.26 - 60.23) |
| 80%  65% | 20-30% | 60.23% (58.24 - 61.35)  53.2% (52.54 - 55.35) | 57.54% (55.63 - 59.68)  49.5% (47.32 - 50.17) |

**Table S8 (a): Total number of MDA rounds and TAS surveys (i.e. proportion of simulations that achieve these rounds) for different baseline prevalences with 80% MDA coverage for the IA drug.**

|  |  | Critical cutoff (target threshold with upper 95% CI) | |
| --- | --- | --- | --- |
| Mean baseline prevalence | Total Number of MDA rounds (TAS surveys) | <7 antigen-positive children (<1%) | <19 antigen-positive children (<2%) |
| 5-10% | 5 rounds (3 surveys)  >= 7 rounds (4 surveys)  7 restart (5 surveys) | 74%  26%  0% | 78%  22%  0% |
| 10-20% | 5 rounds (3 surveys)  >= 7 rounds (4 surveys)  7 restart (5 surveys) | 60%  38%  2% | 62%  34%  4% |
| 20-30% | 5 rounds (3 surveys)  >= 7 rounds (4 surveys)  7 restart (5 surveys) | 41%  53%  6% | 42%  49%  9% |

**Table S8 (b): Total number of MDA rounds and TAS surveys (i.e. proportion of simulations that achieve these rounds) for different baseline prevalences with 65% MDA coverage for the IA drug.**

|  |  | Critical cutoff (target threshold with upper 95% CI) | |
| --- | --- | --- | --- |
| Mean baseline prevalence | Total Number of MDA rounds (TAS surveys) | <7 antigen-positive children (<1%) | <19 antigen-positive children (<2%) |
| 5-10% | 5 rounds (3 surveys)  >= 7 rounds (4 surveys)  7 restart (5 surveys) | 62%  38%  0% | 67%  33%  0% |
| 10-20% | 5 rounds (3 surveys)  >= 7 rounds (4 surveys)  7 restart (5 surveys) | 59%  39%  2% | 57%  37%  6% |
| 20-30% | 5 rounds (3 surveys)  >= 7 rounds (4 surveys)  7 restart (5 surveys) | 37%  54%  9% | 40%  47%  13% |

**Figure S2. Stacked bar plots illustrating the proportion of simulations with the total number of MDA rounds and TAS surveys required (dark grey : more than 7 rounds with restarts, light grey : greater than 7 rounds, white : 5 rounds) across different thresholds with varying baseline prevalences at 80% MDA coverage of the total population alongside the epidemiological outcomes for the IA drug**.


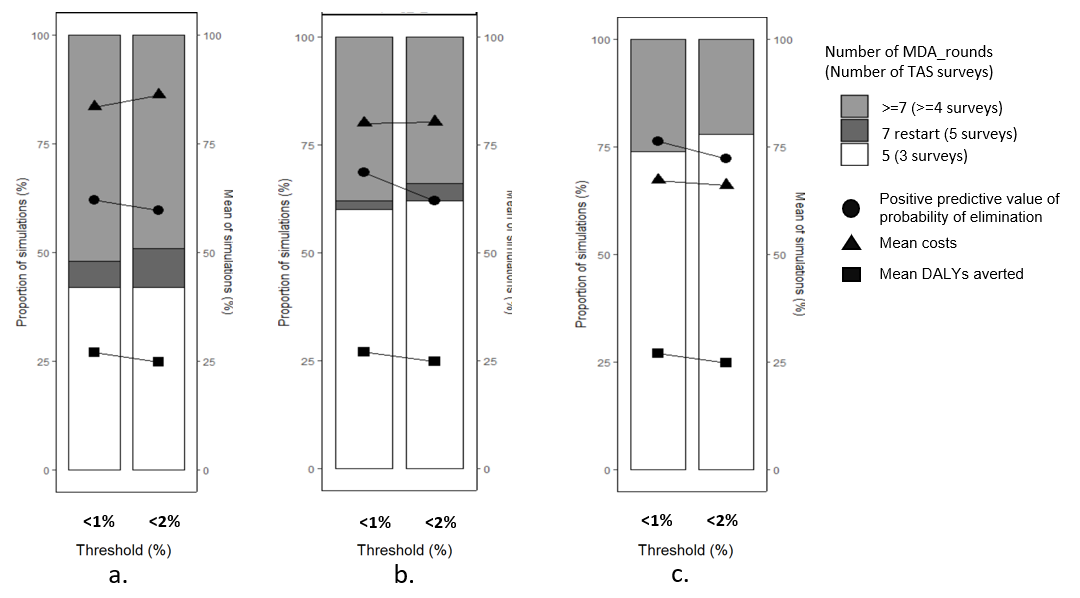


*Figure S2:* *Stacked bar plot shows the trend in the number of rounds of MDA treatment and TAS surveys required for a sample of less than 1700 children aged 6-7 years old with different Ag baseline prevalence (20-30% - left, 10-20% - middle, 5-10% - right) for 80% MDA coverage of the eligible population using IA drug. Light grey represents greater than equal to 7 rounds, dark grey represents 7 rounds with restarts and white represents 5 rounds.  The secondary axis represents the mean of the simulations for the epidemiological outcomes – Circles for positive predictive value of the probability of elimination, Triangles for the mean costs and Squares for the mean DALYs averted for morbidity.*

**Figure S3. Stacked bar plots illustrating the proportion of simulations with the total number of MDA rounds and TAS surveys required (dark grey : more than 7 rounds with restarts, light grey : greater than 7 rounds, white : 5 rounds) across different thresholds with varying baseline prevalences at 65% MDA coverage of the total population alongside the epidemiological outcomes for the IA drug**.


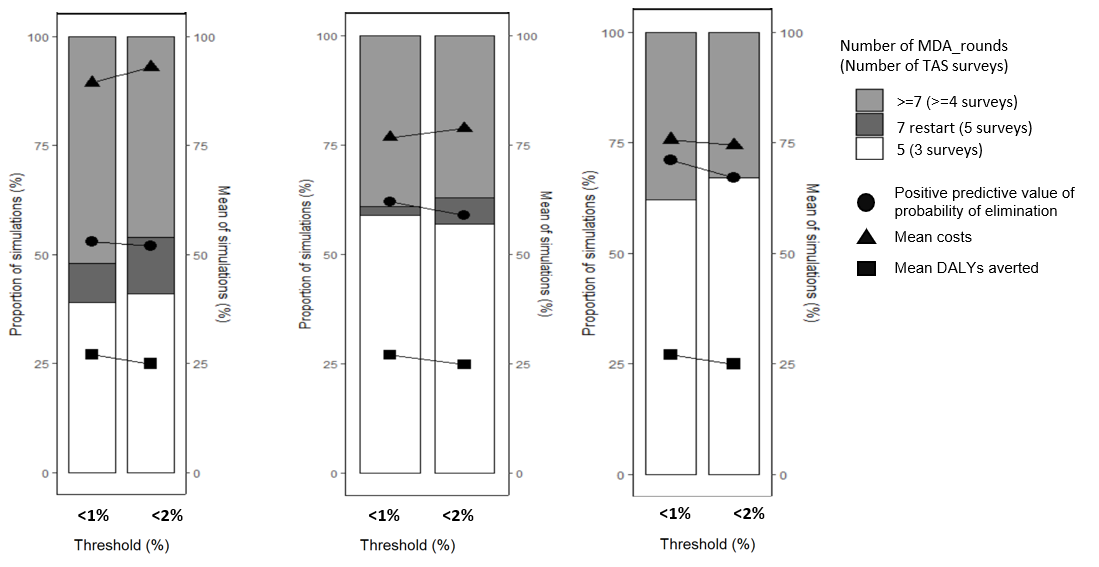


*Figure S3:* *Stacked bar plot shows the trend in the number of rounds of MDA treatment and TAS surveys required for a sample of less than 1700 children aged 6-7 years old with different Ag baseline prevalence (20-30% - left, 10-20% - middle, 5-10% - right) for 65% MDA coverage of the eligible population using IA drug. Light grey represents greater than equal to 7 rounds, dark grey represents 7 rounds with restarts and white represents 5 rounds.  The secondary axis represents the mean of the simulations for the epidemiological outcomes – Circles for positive predictive value of the probability of elimination, Triangles for the mean costs and Squares for the mean DALYs averted for morbidity.*
